# Prior to Peripheral Tolerance, Newly Generated CD4 T Cells Maintain Dangerous Autoimmune Potential: Fas- and Perforin-Independent Autoimmunity Controlled by Programmed Death-1

**DOI:** 10.3389/fimmu.2018.00012

**Published:** 2018-01-24

**Authors:** Kristofor K. Ellestad, Govindarajan Thangavelu, Yohannes Haile, Jiaxin Lin, Louis Boon, Colin C. Anderson

**Affiliations:** ^1^Department of Medical Microbiology and Immunology, University of Alberta, Edmonton, AB, Canada; ^2^Alberta Diabetes Institute, University of Alberta, Edmonton, AB, Canada; ^3^Department of Surgery, University of Alberta, Edmonton, AB, Canada; ^4^Alberta Transplant Institute, University of Alberta, Edmonton, AB, Canada; ^5^Bioceros B.V., Utrecht, Netherlands

**Keywords:** tolerance, autoimmunity, co-stimulation, co-inhibition, lymphopenia, checkpoint inhibitor, cytokine storm, graft versus host

## Abstract

Lymphopenia can result from various factors, including viral infections, clinical interventions, or as a normal property of the fetal/neonatal period. T cells in a lymphopenic environment undergo lymphopenia-induced proliferation (LIP) to fill the available “niche” as defined by peptide–MHC and homeostatic cytokine resources. We recently reported systemic autoimmunity following reconstitution of the lymphoid compartment of Rag1^−/−^ mice with PD-1^−/−^ hematopoietic stem cells or by transfer of thymocytes, but not splenocytes, suggesting that programmed death-1 (PD-1) plays a crucial role in controlling recent thymic emigrants (RTE) and preventing autoimmunity upon their LIP. However, it is unclear whether RTE residing within the periphery of a lymphoreplete host maintain enhanced autoimmune generating potential or if this property only manifests if RTE experience a lymphopenic periphery immediately after export from the thymus. Furthermore, it is unclear which of a variety of T cell effector mechanisms generate pathology when control of RTE by PD-1 is lacking. Herein, we determined that PD-1 is upregulated on CD4 T cells undergoing the natural LIP characteristic of the neonatal period. Newly generated T cells lacking PD-1 maintained an enhanced autoimmune potential even after residence in a lymphoreplete periphery, emphasizing the importance of PD-1 in the establishment of peripheral tolerance. Neither Fas nor perforin-dependent killing mechanisms were required for autoimmunity, while host MHC-II expression was critical, suggesting that LIP-driven autoimmunity in the absence of PD-1 may primarily result from a CD4 T cell-mediated systemic cytokinemia, a feature potentially shared by other autoimmune or inflammatory syndromes associated with immune reconstitution and LIP.

## Introduction

Thymic selection processes can be viewed as the first “filtration” step on the developing T cell repertoire. Although these processes serve to remove the majority of strongly self-reactive T cells from the developing repertoire or convert them to thymic Treg, some self-reactive conventional T cell (Tcon) clones escape (1). This is, for example, evidenced by the ability to induce autoimmune diseases, such as myelin-oligodendrocyte glycoprotein-induced experimental autoimmune encephalomyelitis in mice (2). Peripheral tolerance mechanisms, such as tuning, anergy, deletion, or conversion to peripherally generated Treg (pTreg), are thus important for the establishment and maintenance of immune tolerance and can be viewed as a second filter on the peripheral T cell repertoire.

Aside from the naturally lymphopenic neonatal period associated with immune system development (3), lymphopenia also occurs in a variety of clinical settings. Hematopoietic cell transplantation, T cell depleting therapy for solid organ transplantation, cancer chemotherapy, as well as HIV infection are all associated with lymphopenia of varying severity. In the lymphopenic state, cells undergo a process known as lymphopenia-induced proliferation (LIP), which is facilitated and regulated in its extent by the availability of “resources” for T cells that together define T cell “space” (4). These include peptide–MHC complexes (derived from self or otherwise) that can mediate at least a weak, “tonic” signal through the TCR, as well as homeostatic cytokines, such as IL-7 and IL-15. Regulatory T cells (Treg) (5–13), co-stimulatory molecules such as CD28 (7, 14), as well as molecules with known co-inhibitory activity [e.g., BTLA (15), LAG-3 (16), TGFβRII (17, 18)] can modulate the kinetics of LIP and the maximum size of the T cell compartment. In addition, LIP can promote autoimmune disease and LIP of TGFβRII^−/−^ T cells results in autoimmunity (17).

Programmed death-1 (PD-1), a co-inhibitory receptor expressed on activated T cells, enters the immune synapse upon T cell:APC interaction and is known to recruit the phosphatase SHP2 upon receptor ligation which can dampen proximal TCR signaling cascades (19–21) or co-stimulatory signals through CD28 (22). In contrast to the C57BL/6 PD-1^−/−^ mouse, which displays a relatively mild phenotype characterized by development of a lupus-like disease with spontaneous arthritis and glomerulonephritis upon aging (23), we have shown that reconstitution of the lymphoid compartment of lymphopenic adult Rag1^−/−^ animals *via* transfer of PD-1^−/−^ hematopoietic stem cells (HSC) leads to a rapid, severe, and lethal systemic autoimmune disease soon after the first newly generated T cells, or recent thymic emigrants (RTE), emerge into the periphery (24). The disease is associated with infiltration of CD4 and CD8 T cells into multiple organs, including heart, liver, and kidney, although Rag1^−/−^ K^b−/−^D^b−/−^ animals remain fully susceptible suggesting that MHC-I-restricted CD8 T cells are dispensable for disease. Significantly elevated levels of several pro-inflammatory cytokines and chemokines in serum (24) as well as elevated pro-inflammatory cytokine transcripts in infiltrated organs (25) are also associated with disease. Macroscopically the disease is characterized by kyphosis, cachexia, diarrhea, and skin and ocular lesions. Interestingly, PD-1^−/−^ HSC reconstitution of day 1 Rag^−/−^ neonates results in a drastically reduced incidence of disease (24), suggesting that limited T cell “space” due to small anatomic size (e.g., of lymph nodes) or other factors can limit the aberrant activation of T cells promoted by LIP. Indeed lymph node-deficient Rag^−/−^γc^−/−^ or irradiated LTα^−/−^ hosts were also resistant to disease after PD-1^−/−^ HSC transfer (24). Transfer of PD-1-deficient thymocytes to adult Rag1^−/−^ mice likewise results in autoimmunity; however, transfer of splenocytes from mature PD-1^−/−^ mice does not result in disease. These data suggest that the RTE/newly generated T cell population, which has not yet been subject to peripheral tolerance mechanisms, has greater autoimmune potential than established peripheral T cells and that PD-1 is critically important for controlling their activity during LIP. However, several lines of evidence suggest that newly generated T cells have properties that promote tolerance (26). It is not clear whether newly generated T cells in an adult retain a heightened potential for the generation of autoimmunity after their emergence into the periphery or whether exposure of newly generated T cells to a lymphoreplete environment leads to their rapid tolerization. Herein, we have taken advantage of the Rag2pGFP transgenic (Tg) mouse strain in which GFP is expressed during early T cell development and remains detectable as a marker of newly generated lymphocytes after their emergence into the periphery (27, 28). PD-1^−/−^ peripheral newly generated T cells or established T cells were purified from adult mice and tested for their ability to drive autoimmunity upon transfer into lymphopenic hosts. We found that purified peripheral PD-1^−/−^ newly generated T cells are similar to thymocytes in their ability to drive systemic autoimmunity upon transfer to lymphopenic hosts. Using lymphopenic hosts lacking Fas or MHC-II expression, or PD-1^−/−^ donors lacking perforin expression, we also show that host MHC-II expression is required for disease after PD-1^−/−^ HSC transfer, and that Fas- and perforin-dependent killing mechanisms are dispensable for disease. Taken together, our data suggest that even in a lymphoreplete adult host, peripheral newly generated T cells retain a heightened potential for LIP-driven autoimmunity in the absence of PD-1, which is mediated by CD4 T cells.

## Materials and Methods

### Mice

B6.129S7-*Rag1*^tm1Mom^/J (Rag1^−/−^, Rag^−/−^), B6.Cg-*Foxp3*^tm2(EGFP)Tch^/J (FoxP3^EGFP^, used in the present manuscript as WT), B6.MRL-*Fas*^lpr^/J (Fas^lpr^), C57BL/6-*Prf1*^tm1Sdz^/J (Prf1^−/−^), and B6.129S2-*Ciita*^tm1Ccum^/J (CiiTA^−/−^) mice were purchased from The Jackson Laboratory (Bar Harbor, ME, USA). Days 13–14 gestation pregnant C57BL/6-CD45.1 (B6-CD45.1) mice were purchased from NCI (Frederick, MD, USA). C57BL/6-*Pdcd1*^−/−^ (backcrossed 11 generations to C57BL/6) were originally generated by Prof. T. Honjo and colleagues (23). FoxP3^EGFP^ × *Pdcd1*^−/−^ mice were generated by crossing the above FoxP3^EGFP^ and B6-*Pdcd1*^−/−^ mice and are referred to in the present manuscript simply as PD-1^−/−^. PD-1^−/−^ × Prf1^−/−^ mice were generated by crossing the above PD-1^−/−^ and Prf1^−/−^ mice without any selection for the FoxP3^EGFP^ transgene. Rag^−/−^ × CiiTA^−/−^ mice were generated by crossing the above Rag^−/−^ and CiiTA^−/−^ mice. Fas^lpr^ × Rag^−/−^ mice were generated by crossing the above Fas^lpr^ and Rag^−/−^ mice. B6.Rag2pGFP (Rag2pGFP) mice (27, 28) were kindly provided by Pamela Fink (University of Washington, Seattle, WA, USA). Rag2pGFP × PD-1^−/−^ mice were generated by crossing Rag2pGFP mice with the above B6-*Pdcd1*^−/−^, with screening and selection of breeders for high GFP expression. Marilyn Rag2^−/−^ CD4^+^ anti-HY/I-A^b^ TCR Tg mice (called Marilyn herein) were generated by Lantz and colleagues (29) and were originally obtained from the NIAID exchange program. Cells from Marilyn mice were tracked based on their expression of CD4, CD45.2, and a Vβ6 TCR and lack of CD45.1. Animals were cared for in accordance with the guidelines of the Canadian Council on Animal Care and housed under clean conventional housing conditions at the University of Alberta Health Sciences and Laboratory Animal Services facilities (HSLAS).

### Cell Preparations and Adoptive Transfer Experiments

For experiments involving transfer of thymocytes or peripheral T cells, recipient NK cells were depleted [to avoid potential NK-mediated killing of the input cells (30–32)] by treatment on days −4, −1, and +2 with 0.3 mg per mouse of anti-NK1.1 (PK136) injected intraperitoneally. Thymocytes or splenocytes for injection were prepared by disruption in HBSS (Gibco) + 2% fetal bovine serum (FBS, Sigma-Aldrich) through a 70-µm nylon cell strainer. Cells were centrifuged at ~300 × *g* for 5–10 min at room temperature, and red blood cell lysis was performed by resuspending cells in ACK lysis buffer (150 mM NH_4_Cl, 10 mM KHCO_3_, 0.1 mM Na_2_EDTA) with incubation for 5 min, followed by addition of ~10 volumes of HBSS + 2% FBS, and two cycles of centrifugation at ~300 × *g* for 5–10 min, and resuspension in PBS with no additives. If cells were prepared for further manipulation (e.g., staining and sorting), they were instead washed and resuspended in HBSS + 2% FBS. For experiments involving adoptive transfer of HSC, fetal liver cells (FLC; embryonic days 14–15) were used as a source of HSC. On ice, fetal livers were disrupted by repeated trituration through a 5-mL serological pipet, followed by filtration through a 70-µm nylon mesh filter basket. Cells were then centrifuged at ~300 × *g* for 10 min at 4°C, and resuspended according to their intended further use. FLC were used either fresh or frozen. For immediate use for *in vivo* transfers, FLC were resuspended at 50 × 10^6^ cells/mL in PBS. For freezing, cells were resuspended at 2 × 10^8^ cells/mL in 90% FBS + 10% DMSO, frozen in a −1°C per minute rate-controlled cell freezing apparatus in a −80°C freezer and transferred to the vapor phase of a liquid nitrogen tank for long-term storage. 1.5 × 10^7^ fresh or frozen FLC were transferred intravenously to the indicated recipients. In islet transplantation experiments, female Rag^−/−^ recipients with an established islet transplant received a mixture of 4:1 female B6-CD45.1 and female Marilyn FLC (total of 10–15 × 10^6^ cells). Sex of fetuses was determined by PCR as described (33).

### Definition of Disease and Data Analysis

Macroscopic signs of disease in HSC/thymocyte/peripheral cell recipients included cachexia/weight loss (>15%), kyphosis (hunched appearance), ruffled fur, dermatitis, ocular lesions, and diarrhea. Recipient mice were no longer considered disease free when two or more of the above symptoms were evident, or if mice lost ≥20% body weight. For thymocyte experiments, calculation of weight loss for disease determination was performed relative to weights at day 0 or day 1 relative to cell transfer. For HSC experiments, calculation of weight loss was determined relative to initial weight measurements taken prior to day 30. Unless otherwise indicated, animals were used at 8–16 weeks of age.

### Islet Transplantation

Diabetes was induced in recipient mice *via* treatment with streptozotocin (Sigma-Aldrich) at 185–190 mg/kg. Recipients were considered to be diabetic after two consecutive blood glucose measurements of >20 mM using a OneTouch Ultra glucometer (Lifescan Canada, Burnaby, BC, Canada). Pancreatic islets were isolated from male or female Rag^−/−^ mice as previously described (34). Diabetic recipients were transplanted with 500 islets placed under the kidney capsule and thereafter monitored for return to normoglycemia. All recipients returned to normoglycemia within 48 h of islet transplantation and remained normoglycemic until they were given FLC between 3 and 6 months after islet transplantation; post fetal liver injection some mice with male islets became hyperglycemic (rejected the islet transplant) as described in the Section “Results.”

### Antibodies, Flow Cytometry, and Fluorescence-Activated Cell Sorting (FACS)

For flow cytometric staining and sorting, fluorophore-labeled antibodies against the following markers were obtained from eBioscience (San Diego, CA, USA) unless otherwise indicated: CD4 (RM4-5), TCRβ (H57-597), CD8α (53-6.7), PD-1 (J43), Ki-67 (SolA15), CD44 (IM7), CD19 (1D3), Granzyme B (NGZB), CD45.1 (A20), CD45.2 (104), Vβ6 (RR4-7). Antibodies were used at manufacturer’s recommended concentrations. Flow cytometric staining always used an Fc block cocktail to block nonspecific staining. Fc block cocktail consisted of 3 mL each of normal mouse, rat, and hamster serum, with addition of 0.3 mg of anti-CD16/32 antibody (clone 2.4g2, BioXCell). Fixation and permeabilization were performed using the eBioscience FoxP3 Fixation/Permeabilization buffer kit (Thermo Fisher) according to the manufacturer’s protocols. For cell sorting, a BD Influx cell sorter was used controlled with Spigot software (Beckton Dickinson, Franklin Lakes, NJ, USA). Briefly, for sorting source cells were stained and resuspended in HBSS + 20% FBS + 10 mM HEPES and sorted directly into FBS supplemented with 10 mM HEPES. Standard flow cytometric analysis was performed using a BD LSR II instrument. Flow cytometric data analysis was performed using FlowJo (Treestar software, Portland, OR, USA).

### Statistical Analysis

Statistical analysis was performed using Graphpad Prism software. Details of statistical tests used are provided in figure legends. However, in general, for comparisons of two groups, Student’s *t*-test was used. In cases of comparison of groups with unequal variances, Welch’s correction was applied. Unless otherwise noted, for multiple group comparisons, one-way ANOVA with Tukey’s multiple comparison test was used. Disease onset/incidence was compared by the Kaplan–Meier method. Probability values reported for survival curve comparisons were calculated using the Mantel–Cox method.

## Results

### PD-1 Expression Is Tied to Lymphopenia-Induced Proliferation in Newly Generated T Cells

**Figure 1:**
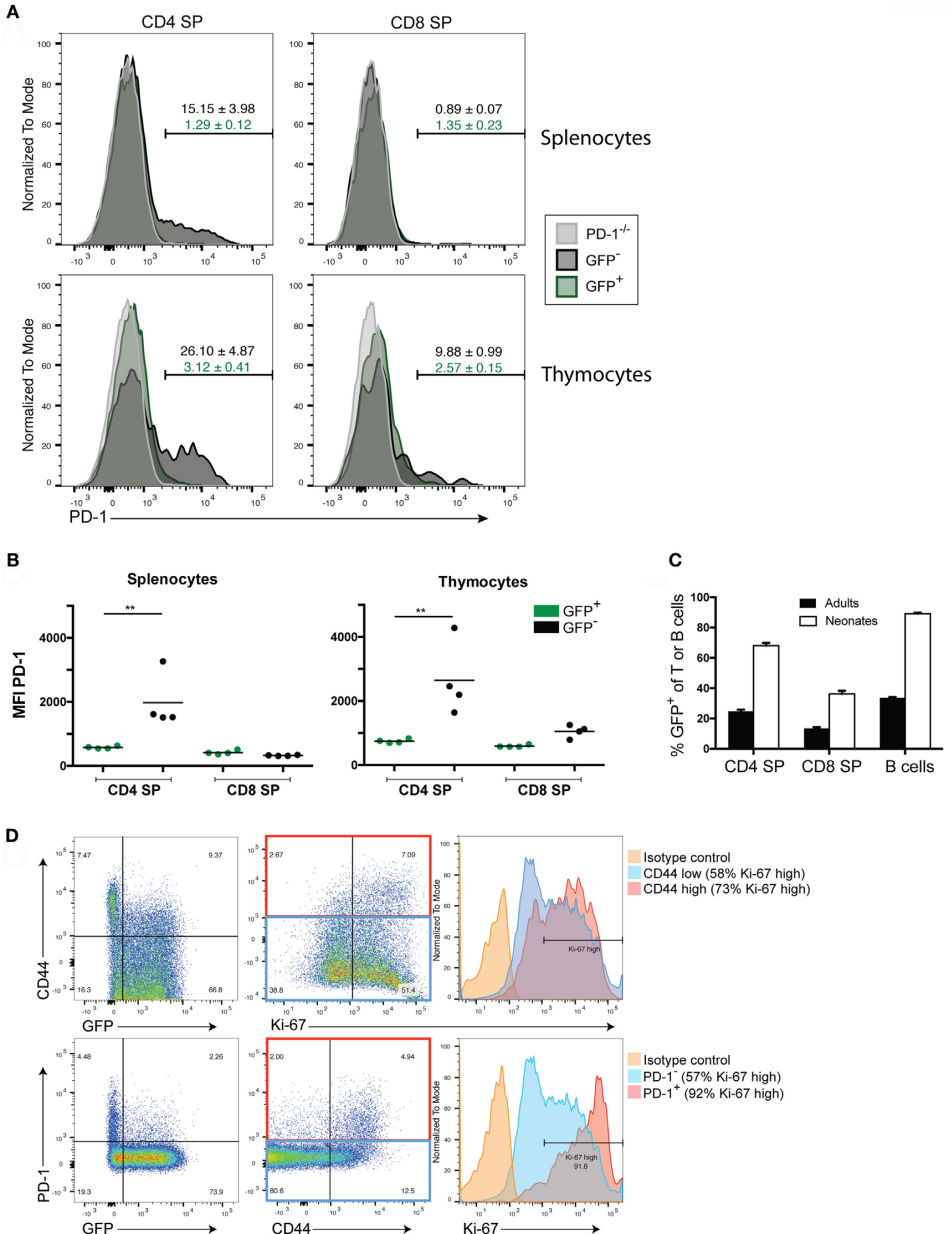
Newly generated T cells in adults lack programmed death-1 (PD-1) expression while in neonates their expression of PD-1 is linked to LIP. **(A)** Representative flow cytometric analysis of splenocytes and thymocytes from 10- to 12-week-old adult Rag2pGFP mice for PD-1 expression among TCRβ^+^ CD4 or CD8 single positive (SP) cells that are GFP^+^ (newly generated T cells) or GFP^−^ (mature cells). T cells from PD-1^−/−^ mice were also stained as a control for background. Values above gates are the group average %PD-1 positive cells within the mature GFP^−^ population (black text) or GFP^+^ population (green text), ±SEM. **(B)** Mean fluorescence intensity (MFI) of PD-1 staining in CD4 or CD8 SP, GFP^+^ or GFP^−^ T cells from Rag2pGFP splenocytes or thymocytes from the experiment depicted in **(A)**. **p < 0.01, one-way ANOVA with Tukey’s multiple comparison test. **(C)** Comparison of percent GFP^+^ cells among peripheral splenocytes in the indicated cell populations in adult vs. neonatal mice ± SEM. *n* = 8 per group. **(D)** Representative (*n* = 3) flow cytometry plots of the indicated markers in splenic TCRβ^+^ CD4 SP T cells from 10-day-old Rag2pGFP neonates. Histogram (right) analyses of Ki-67 on CD44 high or PD-1 high cells and CD44 low/negative cells or PD-1 low/negative cells employed gates as shown in the red and blue rectangles (middle dot plots), respectively. Data presented are from samples that were either permeabilized and stained with antibodies to TCRβ, CD4, CD8, PD-1, CD44, and Ki-67, or non-permeabilized and stained with antibodies to TCRβ, CD4, CD8, PD-1, and CD44 (to allow analysis of GFP).

Programmed death-1 expression appears to be critical for the generation of immune tolerance during LIP of newly generated T cells. We previously showed that newly generated T cells in a lymphopenic setting (i.e., in a lymphopenic recipient of WT HSC) had a high proportion of PD-1 expressing T cells that diminished over time (24). Whether the higher PD-1 expression on newly generated T cells was dependent on exposure to the lymphopenic environment or is an intrinsic property of newly generated T cells even in lymphoreplete mice has not been fully assessed. We, therefore, examined PD-1 expression in the steady state in both TCRβ^+^ thymocytes and peripheral splenocytes from 10- to 12-week-old adult Rag2pGFP animals (Figures 1A,B). Unexpectedly, peripheral newly generated CD4 single positive (SP) splenocytes expressed very little PD-1 while approximately 15% of established or “mature” GFP^−^ CD4 SP T cells were found to be PD-1 positive (Figure 1A). Likewise, mean fluorescence intensity of PD-1 staining was significantly higher in the CD4 SP GFP^−^ cells compared to the GFP^+^ population (Figure 1B). Neither established nor newly generated T cells within the splenic CD8 SP population expressed appreciable levels of this co-inhibitor. Within the thymocyte population, approximately 90% of the CD4 SP and 85% of the CD8 SP were GFP^+^ (data not shown), with the remainder presumably representing mature cells that had recirculated from the periphery back to the thymus, although it is conceivable that at least a subset could represent cells that failed to exit the thymus for longer than 3 weeks post-VDJ recombination (28). GFP^+^ thymic CD4 and CD8 SP cells were slightly enriched for PD-1 positivity (3.1 and 2.6% positive, respectively, Figure 1A) compared to their splenic counterparts (both 1.3%), although this difference was not statistically significant. Similar to what was seen in splenocytes, the thymic CD4 SP GFP^−^ population contained a sizeable population of PD-1^+^ cells (26%), and thus, these more established cells had higher overall PD-1 expression compared to the GFP^+^ newly generated T cell population (Figures 1A,B). Unlike their splenic counterparts, there was a trend toward increased PD-1 positivity and overall PD-1 expression in the thymic CD8 SP GFP^−^ cells compared to the GFP^+^ cells although this difference was not statistically significant (Figures 1A,B).

Thus far, our data indicated that in the steady state, RTE of adult lymphoreplete mice do not express high levels of PD-1 (Figure 1) while adoptive transfer of RTE-generating HSC to lymphopenic mice did lead to high PD-1 expression (24). We, therefore, asked whether heightened PD-1 expression is peculiar to LIP in adoptive transfer or whether it might occur naturally (without adoptive transfer) during the LIP triggered by the lymphopenic state of the neonatal period (3). We examined PD-1 expression on peripheral lymphocytes of 10-day-old Rag2pGFP neonatal mice. More than 97% of thymic CD4 SP cells are GFP^+^ in young Rag2pGFP mice (Figure S1B in Supplementary Material), and all splenic T cells in 10-day-old neonates would be considered to be newly generated based on elapsed time from initial development and, therefore, might also be expected to be GFP^+^. However, a significant percentage (~30% of CD4, ~65% of CD8) of the splenic T cells had lost detectable GFP fluorescence in 10-day-old neonates (Figure 1C). The association between loss of GFP in CD4 T cells and acquisition of a CD44^high^ memory phenotype, as well as an association of CD44 with Ki-67 expression, a marker of cycling cells (Figure 1D, upper panels) suggests that this was due to these cells having undergone multiple rounds of LIP, diluting GFP. Importantly, loss of GFP expression, high CD44 expression, and high Ki-67 expression were all associated with elevated PD-1 expression (Figure 1D, bottom panels): more than 90% of PD-1-expressing CD4 T cells were Ki-67 high. Very few CD8 T cells expressed PD-1 in the neonates at this age and PD-1 expression was not associated with heightened Ki-67 expression in these cells (Figure S1A in Supplementary Material).

Together, these data indicate that while PD-1 is upregulated on T cells following LIP or a period of residency in the periphery (possibly due to encounter with antigen), only very low/barely detectable levels of PD-1 are expressed on newly generated adult T cells. Thus, the heightened PD-1 expression on newly generated T cells seen in lymphopenic HSC recipients is not an intrinsic characteristic of these cells but is LIP-induced. The naturally occurring LIP of the neonatal period also upregulated PD-1 on newly generated CD4 T cells, suggesting PD-1 is involved in establishing tolerance under physiologic conditions (i.e., not just under conditions of cell transfer).

### Steady State Peripheral Newly Generated T Cells in Adult Mice Maintain Heightened Autoimmune Potential Relative to Established T Cells

**Figure 2:**
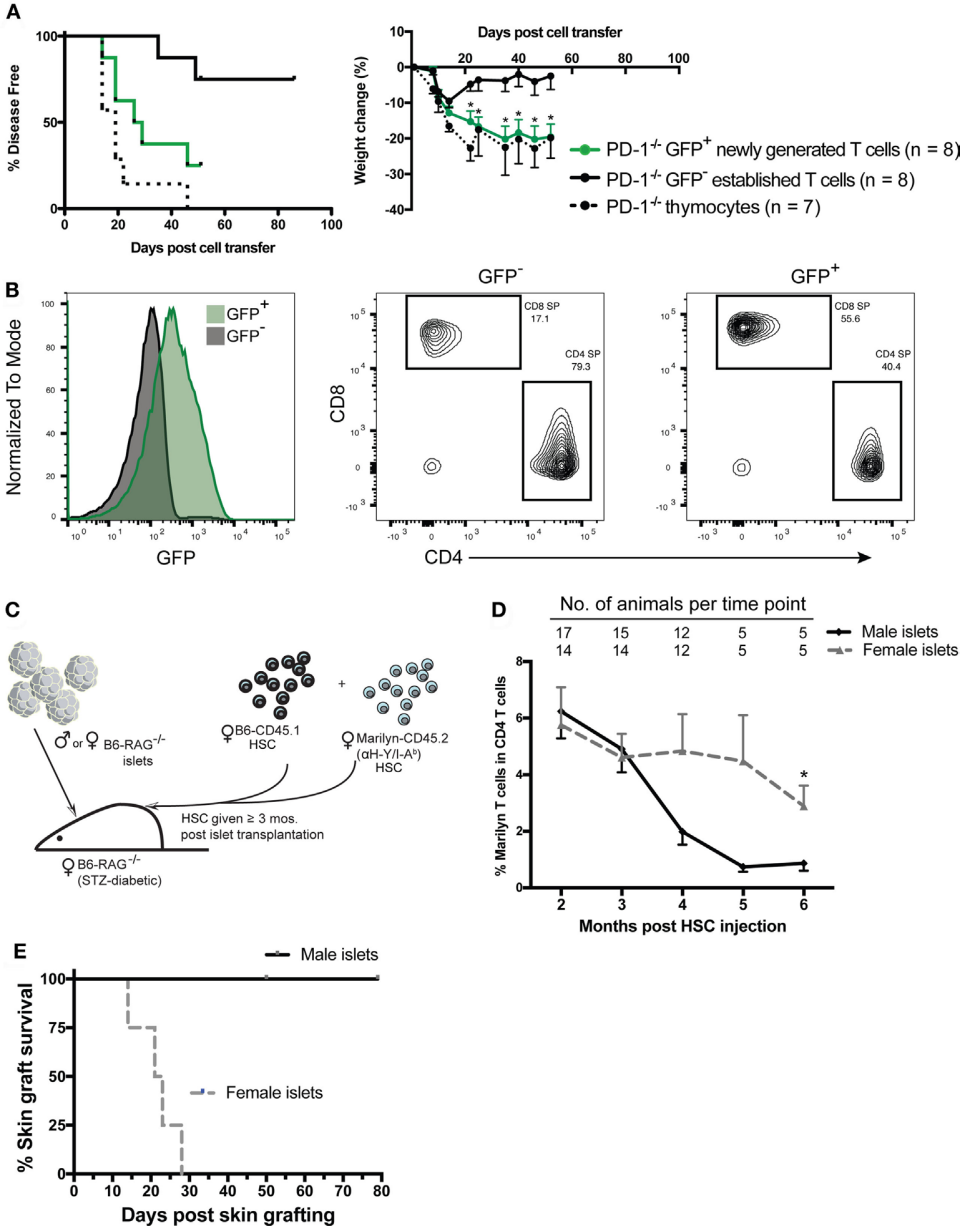
Peripheral PD-1^−/−^ newly generated T cells preferentially drive autoimmunity in lymphopenic recipients. CD4 and CD8 single positive (SP), GFP^+^, and GFP^−^ cells were sorted from ~6-week-old Rag2pGFP × PD-1^−/−^ splenocytes. 1 × 10^6^ sorted CD4 and CD8 SP GFP^+^ or GFP^−^ cells, or unfractionated thymocytes containing an equivalent number of CD4 and CD8 SP cells (~8–9 × 10^6^) were injected into NK depleted adult Rag^−/−^ recipients and mice were monitored for disease symptoms. **(A)** *Left panel*: disease incidence in recipients of thymocytes, GFP^+^ newly generated T cells or GFP^−^ established T cells. Survival curve comparison demonstrated a significant difference between the three groups with *p* = 0.003; PD-1^−/−^ GFP^+^ newly generated cell recipient vs. established cell recipient curves *p* = 0.02; PD-1^−/−^ thymocyte recipient vs. PD-1^−/−^ GFP^+^ newly generated cell recipient curves, *p* = NS. Data are combined from two independent experiments, starting *n* values are indicated in the legend. *Right panel*: weight changes in recipients of indicated cells from two independent experiments ± SEM. Weight observations for which similar time points were available between experiments (±2 days) were combined and used for this analysis. *p < 0.05, GFP^+^ newly generated T cells vs. GFP^−^ established T cells, one-way ANOVA with Tukey’s multiple comparison test. **(B)** Representative GFP expression (left panel) and CD4 and CD8 SP proportions (right 2 panels) in purified cell populations used in **(A)**. **(C)** Diagram of experimental approach using an established minor mismatched transplant as a model peripheral neo-self antigen and anti-donor (HY) TCR transgenic (Tg) Marilyn T cells to track the frequency of CD4 T cells specific to the “neo-self” antigen over time. Male (or control female) islet transplants were allowed to heal ≥3 months into streptozotocin (STZ)-induced diabetic female Rag^−/−^ recipients prior to immune reconstitution *via* transfer of female hematopoietic stem cells (HSC) from CD45 congenic wild-type (B6-CD45.1) and monoclonal TCR Tg mice. **(D)** Frequency of Marilyn T cells (CD4^+^ CD45.2^+^ CD45.1^−^ Vβ6^+^) within total CD4 T cells in the peripheral blood of recipients with male vs. female islets that maintained normoglycemia is shown (mean, SEM, and *n* for each time point is shown). *p < 0.05, Student’s *t*-test. **(E)** Six months post fetal liver cell injection, male skin grafts were transplanted to normoglycemic Rag^−/−^ recipients that had either a male (*n* = 5) or female (*n* = 4) islet transplant and grafts were monitored for rejection. Survival curves are significantly different with *p* = 0.018, Mantel–Cox test.

Our previous studies showed that newly generated T cells exported from the thymus directly into a lymphopenic environment are critically dependent on PD-1 to establish tolerance and prevent autoimmunity. In contrast, PD-1 was not needed to maintain tolerance after transfer of peripheral T cells from PD-1^−/−^ adult animals, which would largely be comprised of established cells. However, it is unknown whether steady-state newly generated T cells in the periphery of immunocompetent adult mice retain this heightened potential for autoimmunity or if instead the peripheral tolerance process is rapid and newly generated T cells are immediately tolerized upon export to a lymphoreplete environment. In order to test the hypothesis that steady-state peripheral RTE/newly generated T cells had increased ability to drive autoimmunity in a lymphopenic host, we generated B6 Rag2pGFP x PD-1^−/−^ mice and purified the GFP^+^ or GFP^−^ T cell populations from splenocytes of adult animals by FACS. Purified cells or thymocytes containing an equivalent number of SP T cells were transferred to Rag^−/−^ recipient animals and disease was monitored. Approximately 20 days after transfer, mice that received either PD-1^−/−^ thymocytes or purified GFP^+^ newly generated T cells began to develop autoimmune disease while the recipients of established cells were relatively spared (Figure 2A, left panel). Similarly, while all recipient mice lost some weight beginning almost immediately after cell transfer until around 2 weeks post-transfer, after this point PD-1^−/−^ established T cell recipients began to regain their lost weight while the thymocyte and newly generated T cell recipients clearly and significantly diverged from the established cell recipients and continued to lose weight (Figure 2A, right panel). Despite the clearly higher propensity of newly generated vs. established cells to drive autoimmunity, it should be mentioned that a low proportion of the GFP^−^ established T cell recipients also met the criteria for disease albeit at a much later time point relative to the other groups (Figure 2A). Approximately, 40% of the purified GFP^+^ newly generated cells were CD4 SP and 56% were CD8 SP (a CD4:CD8 ratio of ~0.7:1), whereas the purified GFP^−^ population contained approximately 80% CD4 SP and 17% CD8 SP (CD4:CD8 ratio ~4.7:1, Figure 2B). While our experiments were designed to test whether the newly generated T cells as a whole have heightened autoimmune potential, the differences in the proportions of CD4 vs. CD8 SP cells in newly generated vs. established T cells could potentially contribute to the differences in their ability to drive disease.

### T Cells with Specificity for a Neo-Self Antigen Can Persist in the Periphery with a Slow Decline in Frequency Over time

The finding that newly generated T cells have a greater potential for autoimmunity than established peripheral T cells appears incongruent with several reported aspects of RTE biology (26) such as reduced effector functions or an increased propensity to convert to pTreg (25, 35, 36) compared with established T cells. These studies raise the question of how newly generated T cells could have a greater capacity to cause autoimmune disease. There are at least two “filters” against self-reactivity, namely central and peripheral tolerance. We surmised that newly generated T cells may have a greater potential for generating autoimmunity because they have yet to pass through the second filter, peripheral tolerance. Autoimmunity caused by newly generated T cells during LIP might be due in part to a greater frequency of T cells with self-specific receptors than are present in the established T cells that have undergone peripheral tolerance mechanisms, such as deletion or conversion to pTreg. In order to explore the concept that potentially dangerous self-reactive cells could persist for a significant length of time in a lymphoreplete periphery post immune reconstitution of lymphopenic mice and be held in check by peripheral tolerance mechanisms without resulting in overt immune pathology, we used the HY antigen expressed by an established graft as a model neo-self antigen (33). We gave mixed Marilyn (anti-male antigen, HY/IA^b^) and WT B6 female HSC to Rag^−/−^ female mice bearing an established islet graft from male or control female Rag^−/−^ donors and monitored subsequent graft rejection and the percentage of cells that were Marilyn over time post immune reconstitution (Figure 2C). We found that reconstitution with a mixture of Marilyn and WT HSC (1:4 Marilyn to WT) led to a small but significant fraction (<30%; 5 of 17) of recipients rejecting the transplant. This rejection appeared to be associated with the appearance in the periphery of a high ratio of Marilyn to WT T cells and earlier export of Marilyn T cells into the periphery compared to WT T cells (data not shown). Nevertheless, the vast majority of recipients tolerated the peripheral male islet transplant. In these male islet graft recipients, we compared the frequency of Marilyn T cells (CD45.2^+^, CD45.1^−^, CD4^+^Vβ6^+^) to recipients of a control female islet graft. The data in Figure 2D show that the male islet transplant, given 3–4 months prior to a 1:4 (Marilyn:WT) mixture of HSC, causes a slow reduction in frequency of neo-self antigen-specific Marilyn T cells. The mice with male but not female islets were tolerant to the male antigen, as they accepted a male skin graft given 4–6 months post HSC (Figure 2E). Thus, peripheral CD4 tolerance was associated with a decreased frequency of neo-self antigen-specific T cells over time, with newly generated T cells having a higher frequency of CD4 T cells specific to the neo-self antigen.

### Host MHC Class II but Not Fas Is Required for Disease upon Transfer of PD-1^−/−^ HSC

**Figure 3:**
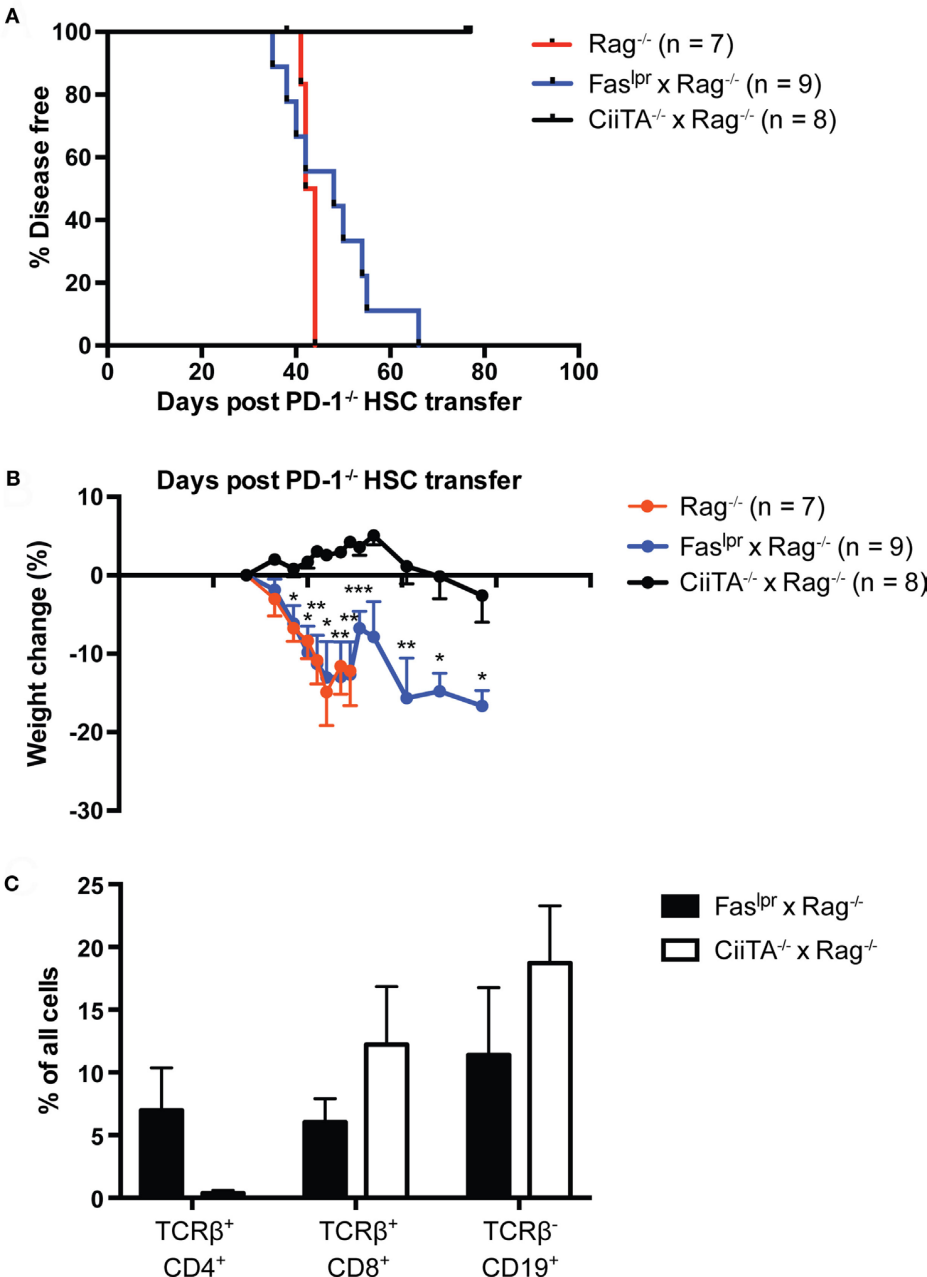
Systemic autoimmunity in lymphopenic PD-1^−/−^ hematopoietic stem cells (HSC) recipients requires host MHC Class II but not Fas. PD-1^−/−^ HSC were transferred to Rag^−/−^, Fas^lpr^ × Rag^−/−^, or CiiTA^−/−^ × Rag^−/−^ recipients and mice were monitored for disease symptoms. **(A)** Kaplan–Meier survival curve analysis of disease incidence among HSC recipients—groups are significantly different with *p* = 0.0002. Data are combined from two independent experiments, with starting numbers per group indicated in the legend. **(B)** Weight changes in recipients relative to day 27 post-transfer, combined from two independent experiments, ±SEM. Weight observations for which similar time points were available between experiments (±2 days) were combined. For each timepoint, only data from groups with *n* > 2 are presented. *p < 0.05, **p < 0.01, ***p < 0.001, Fas^lpr^ × Rag^−/−^ vs. CiiTA^−/−^ × Rag^−/−^, one-way ANOVA with Tukey or Kruskal–Wallis with Dunn’s multiple comparison test (days 27–49) or Student’s *t*-test (day > 51). **(C)** % of all peripheral blood cells that were CD4^+^ or CD8^+^ T cells or CD19^+^ B cells in Fas^lpr^ × Rag^−/−^ and CiiTA^−/−^ × Rag^−/−^ recipients from one experiment in **(A,B)** above, measured at day 46 post-HSC transfer.

We previously showed that adult Rag1^−/−^ K^b−/−^D^b−/−^ mice were fully permissive for the development of autoimmune disease following transfer of PD-1^−/−^ HSC (24); importantly, no perturbations in the time to disease onset or severity were reported compared to Rag^−/−^ hosts, as would be reasonably expected if the disease were CD8 T cell independent. To delineate the importance of CD4 T cells in this disease model, we generated Rag^−/−^ and MHC Class II transactivator-deficient (CiiTA^−/−^) mice, which are largely deficient in MHC-II expression (37). We transferred PD-1^−/−^ HSC to Rag^−/−^ or Rag^−/−^ × CiiTA^−/−^ hosts and monitored the mice for signs of disease. In addition, we examined potential effector mechanisms of disease. Two canonical pathways of T cell killing, namely the FasL-Fas and perforin-dependent pathways, have been described (38). In order to begin to elucidate which, if any, of these pathways are involved in mediating autoimmunity in the PD-1^−/−^ HSC model, we generated a double mutant Fas^lpr^ × Rag^−/−^ mouse, which lacks functional Fas expression (39). While Rag^−/−^ mice developed systemic autoimmune disease around day 44 after transfer, Rag^−/−^ × CiiTA^−/−^ were completely spared (Figure 3A). Similarly, the pronounced weight loss encountered in the Rag^−/−^ recipient group was not seen in the Rag^−/−^ × CiiTA^−/−^ recipients and indeed the latter group actually gained weight for much of the experiment (Figure 3B) despite CD8 T cell and B cell development by day 46 post-transfer (Figure 3C). These data indicate that MHC-II expression in the host is required for disease after PD-1^−/−^ HSC transfer, suggesting disease is dependent on CD4 T cells. Transfer of PD-1^−/−^ HSC to Fas^lpr^ × Rag^−/−^ double mutants revealed that host Fas expression was completely dispensable for the generation of autoimmunity in this model (Figures 3A,B). No statistically significant difference in the survival curves of the Rag^−/−^ and Fas^lpr^ × Rag^−/−^ recipient groups was detected. Similarly, weight loss in Fas^lpr^ × Rag^−/−^ hosts was indistinguishable from that seen in the Rag^−/−^ hosts (Figure 3B). Thus, host Fas expression is not required for disease post PD-1^−/−^ HSC transfer.

### Perforin Expression in the Lymphoid Compartment Is Not Required for LIP-Driven Autoimmunity after Transfer of PD-1^−/−^ Thymocytes

**Figure 4:**
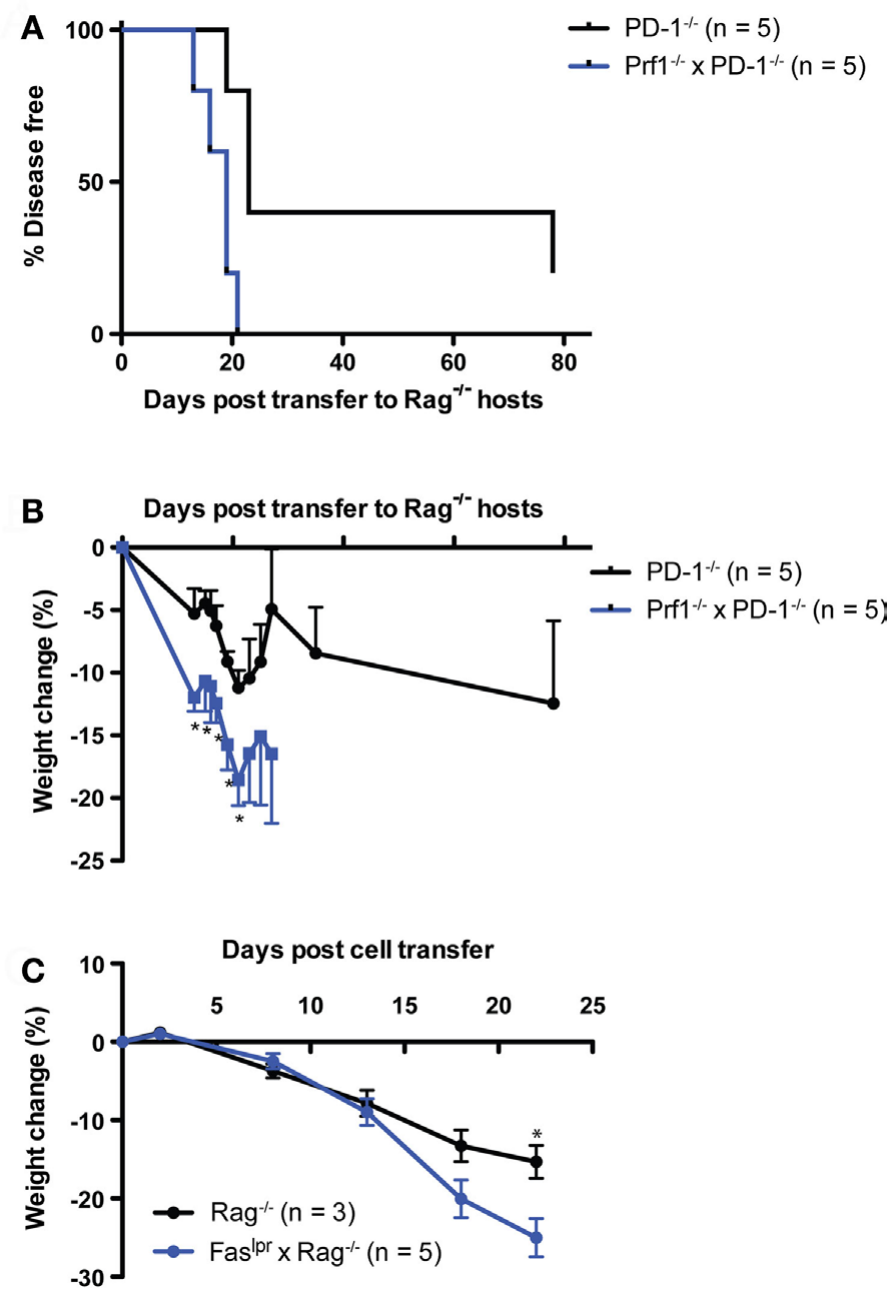
Systemic autoimmunity after PD-1^−/−^ thymocyte transfer to lymphopenic recipients is independent of both perforin and Fas-mediated killing. 10 × 10^6^ Thymocytes from PD-1^−/−^ or Perforin^−/−^ × PD-1^−/−^ (Prf1^−/−^ × PD-1^−/−^) animals were transferred i.v. to NK depleted adult Rag^−/−^ recipients and mice were monitored for disease symptoms. **(A)** Kaplan–Meier survival curve analysis of disease incidence among individual recipients of the indicated thymocytes. Survival curve comparison demonstrated a significant difference between the two groups with *p* = 0.01. **(B)** Weight change among the recipients in **(A)**, ±SEM. Data are from one experiment, starting *n* values are indicated in the legend. **(C)** Splenocytes and inguinal lymph node cells were harvested from three diseased Prf1^−/−^ × PD-1^−/−^ thymocyte recipients from the experiment depicted in **(A)** at day 28 post-transfer, and 20 × 10^6^ cells were transferred i.v. into NK depleted, adult Rag^−/−^ or Fas^lpr^ × Rag^−/−^ recipients. One Fas^lpr^ × Rag^−/−^ recipient received 10 × 10^6^ cells. Weight change among cell recipients, ±SEM. *p < 0.05, Student’s *t*-test. Data are from one experiment with starting *n* values in the legend.

Next, we examined whether perforin expression in lymphocytes was required for disease following transfer of PD-1^−/−^ thymocytes to a lymphopenic host. We generated perforin knockout (Prf1^−/−^) and PD-1^−/−^ mice. The double knockout animals we were able to generate were the F1 progeny of heterozygous crosses and never successfully bred in our facility as homozygous double knockouts (unpublished observations). We transferred thymocytes from the Prf1^−/−^ x PD-1^−/−^ or PD-1^−/−^ mice to Rag^−/−^ recipients and monitored the animals for disease and weight loss. Prf1^−/−^ × PD-1^−/−^ thymocyte recipients all developed autoimmune disease between days 13 and 21 post-transfer, while the PD-1^−/−^ thymocyte recipients had a relatively slightly delayed course of disease (Figure 4A, *p* = 0.01). Furthermore, weight loss (Figure 4B) was significantly greater in the Prf1^−/−^ × PD-1^−/−^ thymocyte recipients, and in general the severity of symptoms, particularly diarrhea, appeared greater in this group compared to PD-1^−/−^ thymocyte recipients. In addition to these findings, we also performed two experiments to question whether Prf1^−/−^ was required for disease in the HSC transfer model. In the first experiment, Prf1^−/−^ × PD-1^−/−^ T cell depleted bone marrow cells were transferred to Rag^−/−^ hosts. In the second, Prf1^−/−^ HSC were transferred to Rag^−/−^ hosts treated intraperitoneally with 200 μg/mouse monoclonal blocking anti-PD-1 antibody (clone J43) every 2 days from day 25 until termination. In both of these experiments, recipients developed autoimmunity (data not shown) further supporting the notion that perforin expression in T cells is not required for disease in this model.

Because of the potential for functional redundancy between the Fas–FasL and perforin-dependent killing pathways, we also tested whether adoptive transfer of cells from diseased Rag^−/−^ recipients of Prf1^−/−^PD-1^−/−^ thymocytes to Fas^lpr^ × Rag^−/−^ hosts would result in the development of autoimmunity. Indeed, splenocytes + lymph node cells from the diseased Prf1^−/−^PD-1^−/−^ thymocyte recipients generated robust development of autoimmunity when transferred either to Rag^−/−^ or Fas^lpr^ × Rag^−/−^ recipients (disease onset: day 18, 22, >22 vs. 13, 18 × 3, 22, respectively; *p* = NS). Although both recipient groups lost weight, by day 22 post-adoptive transfer the Fas^lpr^ × Rag^−/−^ hosts had lost significantly more weight than the Rag^−/−^ recipients (25 vs. 15%, Figure 4C), suggesting that Fas in the host may actually play an immunoregulatory role. Taken together, these data show that neither perforin expression in T cells nor Fas expression in the host are required for LIP- and newly generated T cell-driven autoimmunity in the context of PD-1 deficiency.

## Discussion

Mechanisms of T cell homeostasis function to maintain a diverse repertoire of sufficient size for effective immune surveillance of the host. Reconstitution of the T lymphocyte compartment of a lymphopenic host by HSC transplant or transfer of T cells results in LIP as the cells expand to fill the available niche defined by available peptide–MHC (pMHC) and cytokine “resources.” The relative abundance of resources in a host with respect to the numbers of competitors for those resources can be considered as a way to define the “severity” of lymphopenia in that host or its “LIP-potential” (40). Importantly, LIP is strongly associated with the promotion of autoimmune or inflammatory disease (5, 13, 17, 24, 40–49). Such promotion of autoimmunity may result from potentiation of TCR signaling by high concentrations of homeostatic cytokines like IL-7 and IL-15 (50, 51) and/or unimpeded access to self-pMHC due to decreased competition from other Tcon or Treg. Thus, perturbations which would be expected to increase the LIP potential of a host, such as decreased Treg, increased homeostatic cytokines, or decreases in co-inhibitory signals would be expected to increase the potential for such autoimmunity, and *vice versa*. Newly generated T cells during LIP appear particularly predisposed to the generation of autoimmunity when PD-1 mediated co-inhibitory signals are compromised (24). Newly generated T cells have not been subjected to peripheral tolerance mechanisms and thus may contain an increased proportion of strongly self-reactive cells compared to established T cells that arose in a host with low LIP potential upon initial seeding of its peripheral T cell compartment (i.e., during the neonatal period) and were tolerized. Indeed, in the current studies, we showed that a high frequency of CD4 T cells specific to a peripheral neo-self antigen can persist for an extended period in a lymphoreplete adult host, declining slowly with time without overt immune-mediated pathology (i.e., lack of rejection of the neoantigen expressing graft; Figures 2C–E). In addition, we have recently shown that PD-1 contributes to control of tonic pMHC signaling, which may tune signaling thresholds to establish a general T cell homeostasis needed for peripheral tolerance (52). Altogether our data suggests that PD-1 is more critical for control of newly generated CD4 T cells because of increased self-specific T cells in this population as well as a need to set response thresholds to tonic pMHC signals in all CD4 T cells. Although this view is consistent with recent findings from Jiang and colleagues, in which they showed PD-1 was important in reducing the number of high-affinity self-specific CD4 T cells (53), their conclusion differs substantially from our own. While they concluded that PD-1 is needed to maintain tolerance, both their data and ours are instead consistent with the view that PD-1 is needed to establish tolerance, with PD-1 playing relatively little or no role in the maintenance of tolerance once it is established. On the other hand, much of the published literature describing newly generated T cells/RTE suggests that they have functional properties geared toward promotion of tolerance (26). However, we found that newly generated PD-1^−/−^ T cells isolated from the periphery of adult animals were indeed capable of generating autoimmune disease (particularly cachexia, kyphosis, and diarrhea) similar to PD-1^−/−^ thymocytes, and much more efficiently than established T cells which contained approximately 1.3% contaminating GFP^+^ cells (Figure 2A). These data indicate that steady state newly generated T cells have a dangerous autoimmune potential even when their initial export from the thymus is into a lymphoreplete environment.

Tolerance first becomes established in mice during the neonatal period. Compared to adult Rag^−/−^ mice, we consider that prior to/during initial seeding of the lymphoid compartment neonatal mice would, by virtue of anatomic size (small lymph nodes, reduced overall resources) and competition by specialized populations of innate lymphoid cells for IL-7 (54) have significantly lower LIP potential. This is supported by our finding that neonatal Rag^−/−^ hosts rarely developed severe autoimmunity after reconstitution with PD-1^−/−^ HSC (24). A lymphoreplete adult WT host on the other hand would be considered to have lower LIP potential than either an adult Rag^−/−^ or a WT neonate (i.e., the ranking of LIP potential would be adult Rag^−/−^ > neonatal WT > adult WT) (40). We hypothesize that the mild but significant LIP potential in neonates creates a situation in which establishment of tolerance in the nascent T cell population by PD-1 is important, and the absence of PD-1-mediated control of neonatal LIP may set the stage for the lupus-like autoimmunity that manifests later in life in PD-1^−/−^ animals.

Although PD-1 is critical for tolerance in newly generated T cells, and PD-1 was expressed on neonatal T cells undergoing LIP, we did not, in the steady state, detect PD-1 expression above background on the peripheral newly generated T cell population of adult Rag2pGFP mice and only low expression in thymic GFP^+^ T cells (Figure 1). In contrast, both the peripheral CD4 SP and thymic CD4 and CD8 SP established (GFP^−^) T cell population contained sizeable populations that robustly expressed PD-1. Previous data from our lab assessing PD-1 expression in steady state RTE (i.e., in WT lymphoreplete mice) used CD24 as the marker to define RTE and the data suggested RTE have higher PD-1 (24). However, based on our current data with the Rag2pGFP mouse and the fact that only a small fraction of newly generated T cells expressed CD24 and established T cells can also express CD24, we conclude that steady-state adult newly generated T cells do not have increased PD-1. Together with our finding that PD-1 can regulate LIP and the response to tonic pMHC signals (25, 52), these observations suggest that PD-1 is upregulated during LIP as a negative feedback mechanism. Finally, it is worth noting that because a significant portion (10–15%) of thymic T cells are established cells based on lack of GFP expression, consistent with the previously described ability of peripheral T cells to recirculate back to the thymus (55), the use of the term “recent thymic emigrants” to describe GFP^+^ cells in the Rag2pGFP Tg model as is common in the literature is somewhat imprecise as presumably some cells emerging from the thymus in an adult are also GFP^−^. Therefore, although all peripheral GFP^+^ cells are RTE, all RTE are not necessarily GFP^+^.

One potential explanation for our finding that purified peripheral newly generated T cells efficiently caused disease upon transfer to lymphopenic hosts while established cells did not is that the relative proportions of CD4 and CD8 T cells differed significantly between these populations. Based on the present data including the lack of disease in CiiTA^−/−^ hosts despite generation of CD8 T cells and B cells (Figure 3C) and our previous findings that MHC Class I-deficient lymphopenic hosts were fully disease permissive, we conclude that CD4 T cells in this setting are the key effectors of autoimmune pathology. Given that the purified established T cell population contained a greater frequency of CD4 vs. CD8 T cells, their inability to drive disease (and conversely the ability of GFP^+^ PD-1^−/−^ newly generated cells to do so) cannot be explained by insufficient numbers of CD4 T cells.

While CD4 T cells are most commonly considered as “helpers” of the immune response, numerous studies have suggested that they can in some circumstances acquire cytolytic effector function *via* upregulation of killing mechanisms typically associated with CD8 cytotoxic T lymphocytes such as the perforin and Fas-ligand (FasL) pathways (56). Our finding that neither Fas^lpr^ × Rag^−/−^ recipients of PD-1^−/−^ HSC, nor recipients of Prf1^−/−^ × PD-1^−/−^ thymocytes were spared from disease (Figures 3 and 4A,B) suggests that both of these canonical T cell effector pathways are dispensable for LIP-driven autoimmunity in the setting of PD-1 deficiency. Furthermore, our finding that disease in Prf1^−/−^ × PD-1^−/−^ thymocyte recipients was exacerbated compared to PD-1^−/−^ thymocyte recipients (Figures 4A,B) suggests that perforin-dependent effector pathways may actually play an immunoregulatory role during LIP. This is perhaps not surprising given that perforin has been reported to be important for contraction of the CD8 population following infection (57, 58), and as a mediator of suppression by Tr1 cells (59). Similarly, weight loss of increased severity in the Fas^lpr^ × Rag^−/−^ compared to Rag^−/−^ recipients of adoptively transferred Prf1^−/−^ PD-1^−/−^ cells (Figure 4C) suggested that Fas may also play an immunoregulatory role during LIP. This might also be anticipated given the lymphoproliferative disease characteristic of the Fas and Fas-ligand-deficient *lpr* and *gld* mice (60) which is partially attributable to defects in Fas-mediated killing of antigen-presenting cells (61). The latter experiment also ruled out functional redundancy between Fas–FasL and perforin-dependent killing pathways. We did consider the possibility that Granzyme B produced by T cells activated during LIP might act independently of perforin, for example through the mannose-6-phosphate receptor (62). However, examination of Granzyme B expression in WT vs. PD-1^−/−^ HSC recipients at the peak of disease (day 45) demonstrated no significant difference in splenic CD4 or CD8 T cell populations although a small trend toward increased expression in PD-1^−/−^ HSC recipient CD8 T cells was noted (Figure S2 in Supplementary Material).

The broad upregulation of a number of pro-inflammatory cytokines in PD-1^−/−^ HSC recipients (IFN-γ, IL-13, TNF-α, IP-10, MIG, MCP-1, VEGF) (24) along with the lack of requirement for either perforin- or Fas-dependent killing pathways for autoimmunity in this model suggest that the immune pathology seen is primarily a CD4 T cell and “cytokine-storm” dependent phenomenon. Several similarities exist between the LIP-driven autoimmunity described herein and certain clinical syndromes, such as immune reconstitution inflammatory syndrome (IRIS) in HIV patients experiencing a rebound of the CD4 T cell compartment after treatment with antiviral drugs (44, 63) or in chronic GVHD post-allogeneic bone marrow transplantation (64). Notably a mouse model of IRIS was recently described in which transfer of purified CD4 T cells to *Cryoptococcus*-infected lymphopenic mice yields weight loss and systemic inflammatory disease associated with cytokine dysregulation (45). In addition, the striking increases in the chemokines IP-10 and MIG described previously in our model (24) are also observed in clinical chronic GVHD (65). Indeed, the term “cytokine-storm” was originally used to describe the syndrome of cytokine dysregulation (particularly IL-6, IL-1, and TNF-α) which is associated with and greatly contributes to GVHD pathology (66, 67). One could make the argument that our model of LIP-driven autoimmunity after transfer of PD-1^−/−^ newly generated T cells to a lymphopenic host might actually be viewed as a model of syngeneic GVHD, similar to the autoimmunity that occurs when cyclosporine A (CsA) is discontinued post bone marrow transplantation. However, broad-spectrum antibiotics prevented CsA-induced syngeneic GVHD (68) but did not prevent disease in our model (25). Instead, the stimulus for PD-1-deficient RTE appears to involve autoantigens, potentially including the low-affinity interactions with self-peptide MHC that otherwise generate only tonic survival signals (40). Thus, beyond the insights it offers into the role of co-inhibitory molecules in establishing tolerance, further characterization of this model may lead to translatable insights to treat cytokine-driven systemic autoimmunity and inflammatory disease including after therapies involving immune reconstitution.

## Ethics Statement

This study was carried out in accordance with the recommendations of the Canadian Council on Animal Care. The protocol was approved by the University of Alberta Health Sciences Animal Care and Use Committee.

## Author Contributions

KE designed, performed research and data analysis, and wrote and critically edited the manuscript. GT, YH, and JL designed and performed research and data analysis and critically edited the manuscript. LB provided reagents and critically edited the manuscript. CA designed research, performed data analysis, and critically edited the manuscript.

## Conflict of Interest Statement

The authors declare that the research was conducted in the absence of any commercial or financial relationships that could be construed as a potential conflict of interest.

## Abbreviations

LIP, lymphopenia-induced homeostatic proliferation; HSC, hematopoietic stem cells; pTreg, peripherally generated regulatory T cell; Treg, regulatory T cell; SP, single positive; RTE, recent thymic emigrants; pMHC, peptide–MHC; Tcon, conventional T cell; PD-1, programmed death-1; MFI, mean fluorescence intensity; WT, wild type.
