# Supplementary figures and images for: Prior to Peripheral Tolerance, Newly Generated CD4 T Cells Maintain Dangerous Autoimmune Potential: Fas- and Perforin-Independent Autoimmunity Controlled by Programmed Death-1
Source: Front Immunol. 2018 Jan 24;9:12. doi: 10.3389/fimmu.2018.00012 (PMC5787554; doi:10.3389/fimmu.2018.00012)

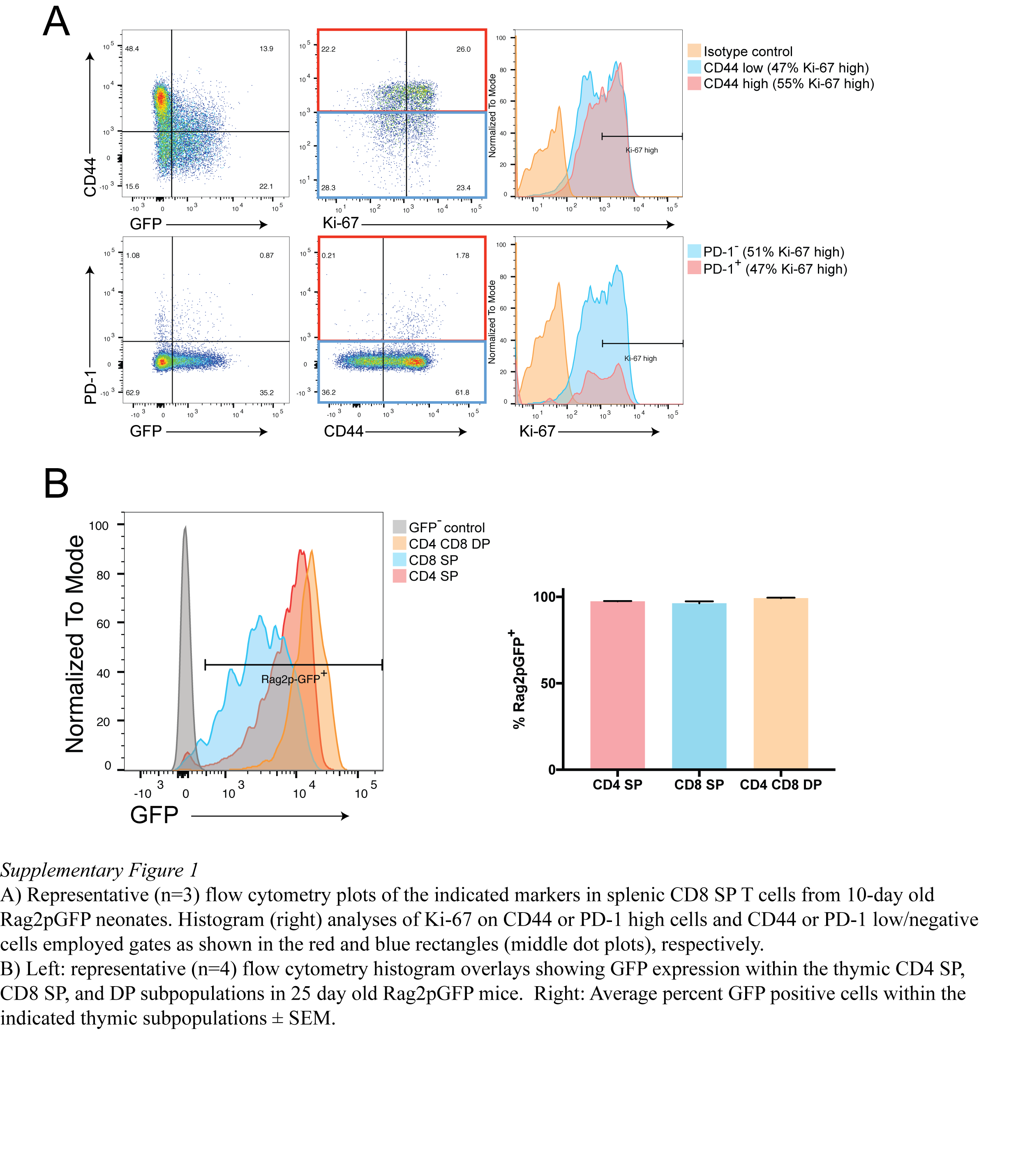

Supplement: Supplementary file 1 [file Image_1.TIF]

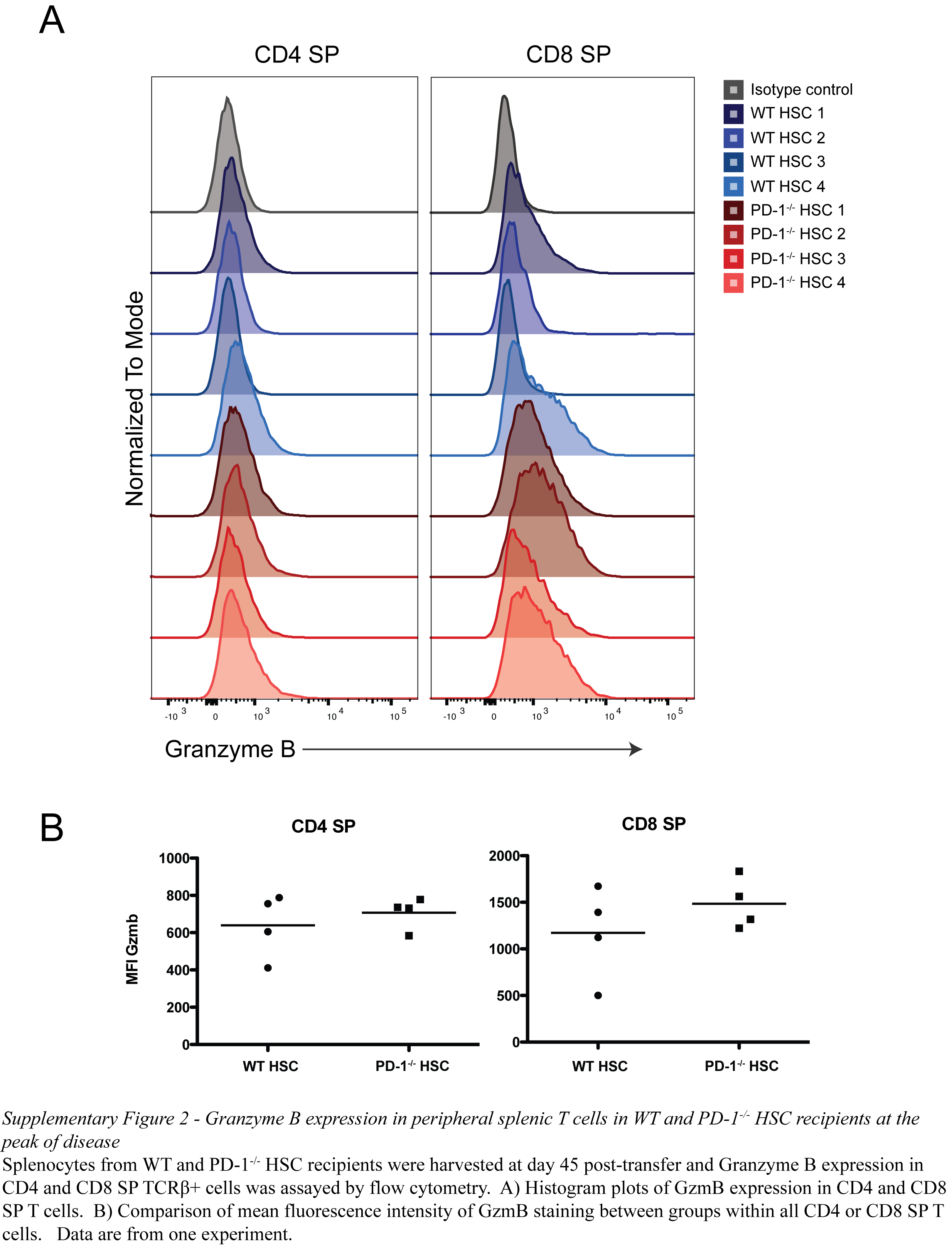

Supplement: Supplementary file 2 [file Image_2.TIF]
